# Nabilone administration in refractory chronic diarrhea: a case series

**DOI:** 10.1186/s12876-019-1024-y

**Published:** 2019-06-25

**Authors:** Lanfranco Pellesi, Maria Chiara Verga, Nicola De Maria, Erica Villa, Luigi Alberto Pini, Simona Guerzoni

**Affiliations:** 1 Medical Toxicology and Headache Center, University of Modena and Reggio Emilia, via del Pozzo 71, 41124 Modena, Italy; 2 Gastroenterology Unit, Department of Internal Medicine, University of Modena and Reggio Emilia, Modena, Italy; 3 Centre for Neuroscience and Neurotechnology, University of Modena and Reggio Emilia, Modena, Italy

**Keywords:** CB1 receptor, Cannabinoids, Nabilone, Chronic diarrhea, Gastrointestinal disorders, Refractory, Case series

## Abstract

**Background:**

Daily cannabis assumption is currently associated with several physical and mental health problems, however in the past it was prescribed for a multitude of symptoms, including vomiting, abdominal pain and diarrhea. Through the years, the endocannabinoid system has been recognized in the homeostatic mechanisms of the gut, as well as in the physiological control of intestinal motility and secretion. Accordingly, cannabinoids may be a promising therapy against several gastrointestinal conditions, such as abdominal pain and motility-related disorders.

**Case presentation:**

We retrospectively analysed the efficacy and safety of a CB1-receptor agonist administered in six patients with refractory chronic diarrhea, between April 2008 and July 2016. After three months of therapy, oral nabilone improved the health of nearly all patients, with visible improvements in reducing diarrheal symptoms and weight gain. Most of the benefits persisted through the three-month follow-up. Only one patient interrupted the treatment after one month, due to severe fatigue and mental confusion; the symptoms disappeared in the follow-up period.

**Conclusions:**

These findings encourage the study of cannabinoids acting on CB1 receptors in chronic gastrointestinal disorders, especially in refractory chronic diarrhea, offering a chance for a substantial improvement in the quality of life of selected patients, with a reasonable safety profile.

## Background

Cannabis is the most abused illicit drug in the world [1]. In the past, it played a strong and prominent role in medicine. Until 1941, when cannabis preparations were taken off the *United States Pharmacopeia and National Formulary*, it was prescribed for a multitude of symptoms, including nausea, vomiting, abdominal pain and diarrhea [2, 3]. Even though the consumption of cannabis is currently associated with major health issues, such as an increase in respiratory diseases, psychosis and suicidal ideations [4], the medical use of its derivatives continues to increase worldwide. At the time of this writing, several European nations and more than 30 United States territories allow marijuana for medical purposes, although their approaches are significantly different. Moderate evidence now supports the use of cannabis for the treatment of chronic pain and spasticity, and improvements have been observed in nausea and vomiting due to chemotherapy, anorexia in HIV infection, sleep disorders and Tourette syndrome [5].

Among the active ingredients of cannabis, cannabinoids are the most studied in the medical literature. Δ [9]-tetrahydrocannabinol (Δ [9]-THC) is the most familiar of them, and the responsible for the psychotropic activity. Once absorbed by the human body, several cannabinoids interact with the endocannabinoid system (ECS), a physiologic system involved in establishing and maintaining homeostasis of different organs and body systems. ECS is composed by endogenous ligands, known as endocannabinoids, by G protein-coupled cannabinoid receptors 1 and 2 (CB1 and CB2) and the enzymes involved in endocannabinoids turnover [6–8]. It interacts within its own pathways, as well as with all major inflammatory and painful pathways, including endorphins, enkephalins, transient receptor potential cation channels (TRPV) and peroxisome proliferator-activated receptors (PPARs) [9]. ECS components are in the human brain and in the peripheral nervous system, but they are also detectable outside the brain, e.g. on immune cells, in the gastrointestinal (GI) tract and other organs [10].

In recent years, the knowledge of the ECS and its role in the gut has grown rapidly. The actions of the ECS appear to be largely homeostatic, contributing to the gut protection from inflammation and regulating GI motility [11–13]. CB1 receptors are expressed in the normal human colon and in the enteric nervous system (ENS), regulating neurotransmitters release, intestinal smooth muscle tone and peristalsis [14], whereas CB2 receptors were found in the lamina propria and in the ENS [15]. Rimonabant, an inverse agonist of CB1 receptors, was able to increase colonic motility in mice [14], while the agonist activity of dronabinol inhibited gastric emptying and colonic motility in healthy humans [16]. Furthermore, the endocannabinoid signalling is intensified during intestinal inflammation; CB2 receptors are up regulated in colonic mucosal samples from inflammatory bowel disease (IBD) patients [17] and JWH-133, a CB2 receptor agonist, inhibits the increase in GI transit elicited by an intraperitoneal injection of lipopolysaccharides [18]. These results are consistent in over than 10% of patients with IBD, who confirmed that they had used cannabis as self-medication at least once in their life [19], and most of them found that marijuana was very helpful to relieve abdominal pain and diarrhea [20, 21]. Accordingly, cannabinoids acting on CB1 and CB2 receptors might have beneficial effects for patients with symptoms of abdominal pain, inflammation and hypermotility [22–24]. The latter often coexist in chronic diarrhea. Chronic diarrhea is defined as a symptom with at least three bowel movements per day, with a faecal weight of more than 200 g/day, lasting for at least four weeks [25]. It is due to many non-specific causes; as a result, patients take nonspecific antidiarrheal therapy (such as opiates, intraluminal pro-absorptive agents or glucocorticoids) for several months, and do not solve their problem.

## Case presentation

**Fig. 1:**
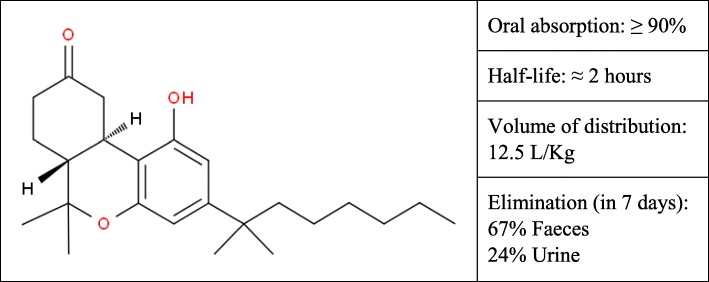
Pharmacological properties of nabilone reported by Lemberger et al. [26]. Chemical structure of nabilone is reproduced by permission of The Royal Society of Chemistry [27]

We present the course of six patients with refractory chronic diarrhea, treated with a synthetic CB1-receptor agonist. The baseline characteristics of patients are presented in Table 1. The reasons for chronic diarrhea are manifold and are explained in the subsequent description of the cases. All patients were previously treated with etiologic and symptomatic drugs, without benefits. The pharmacological properties of the drug, nabilone, are summarized in Fig. 1. They are deduced from the work of Lemberger et al. [26]. The drug was administered orally in the hospital setting, as a compassionate drug, for three months. For the duration of the treatment and the following three months, all patients were periodically monitored by medical toxicologists due to the possible occurrence of adverse events and/or addictive behaviours.

**Table 1:** Baseline characteristics of patients with refractory chronic diarrhea

Patient	Sex	Age (years)	Weight (Kg)	Primary disease	Duration of chronic diarrhea	Daily bowel movements
1	F	45	50	Crohn’s disease	5 months	6
2	F	54	42	Crohn’s disease	12 years	10
3	F	75	38	Pancreatic cancer treated with spleno-pancreatectomy	25 months	3
4	M	40	46	Sequelae of an extended mesenteric thrombosis	2 months	8
5	F	71	45	Systemic sclerosis	7 months	10
6	F	47	38	Short bowel syndrome	11 months	8

### Patient 1

She was a 45-year-old, white female, with a history of intestinal obstruction and ileocecal resection occurred in January 2006, followed by a histologic diagnosis of Crohn’s disease. In May 2007, she was admitted to the hospital for a reactivation of the inflammatory disease, the colonoscopy revealed an anastomotic recurrence, which was treated with steroids (prednisone, 25 mg daily). A daily diarrhea appeared in November 2007; at the same time, the patient suffered from chronic headache with non-steroidal anti-inflammatory drugs (NSAIDs) abuse, and chronic gastritis. A new admission was performed in April 2008 for a worsening of chronic diarrhea and headache (weight = 50 Kg, haemoglobin or Hb = 7.9 g/dl, haematocrit or Ht = 25.3%, C-reactive protein or CRP = 1.33 mg/dl, Erythrocyte Sedimentation Rate or ESR = 43 mm/h, Crohn’s Disease Activity Index or CDAI = 157). On admittance, the patient complained of abdominal pain and six bowel movements per day, with watery stools. Colonoscopy showed a narrow stenosis of the anastomosis, with large mucosal erosion, that did not allow the instrument progression (lack of visualization of the ileal mucosa). The patient was treated with steroids (prednisone, 50 mg daily) and azathioprine (100 mg daily). Due to the failure of previous therapies (including rifaximin), the patient started taking nabilone (1 mg/day) to control both diarrhea and chronic headache. Concurrent medications included mesalazine (1500 mg/day), lansoprazole (30 mg/day), sodium valproate (600 mg/day), prednisone (50 mg/day), citalopram (40 mg/day), azathioprine (100 mg/day), tramadol (15 drops as needed) and clonazepam (15 drops/day). After three months of treatment, nabilone was discontinued, the patient had one bowel movement per day, without blood or mucus in the stools (weight = 52 Kg, Hb = 9 g/dl, CRP = 1.69 mg/dl, ESR = 19 mm/h, CDAI = 82); no colonoscopy was performed. Three months after the end of therapy, the patient had 2 bowel movements per day, no abdominal pain, diarrhea and/or blood in the stools (weight = 52 Kg, Hb 11.7 g/dl, Ht = 34,3%, CRP = 0.23 mg/dl, ESR = 10 mm/h). The patient had no further diarrhea episodes, but she is still suffering from chronic headache, despite numerous therapeutic changes.

### Patient 2

She was a white female, 54-year-old, with a history of Crohn’s disease since the age of 22, when an acute appendicitis surgery happened. She had undergone other abdominal surgeries, in 1986 (removal of 45 + 5 cm of bowel and ileocecal resection, latero-lateral anastomosis), in 1990 (resection of 36 cm of residual bowel, right colon and transverse colon, end-to-end anastomosis), and in 2004 (ileo-colonic resection of 20 cm and surgical removal of an abscess). The first symptoms of diarrhea occurred after the last surgical operation, the patient started to use loperamide tablets (2 mg), up to four daily. In 2010, an entero-cutaneous fistula appeared; it was successfully treated with adalimumab (discontinued due to mild hypertransaminasemia) and hyperbaric therapy. In July 2015, a new surgical scraping was performed because of the re-emergence of the fistula, along with an ileo-colonic anastomosis resection and ileostomy. The patient received a histologic diagnosis of rectal adenocarcinoma, treated with chemotherapy and radiotherapy. In January 2016, she returned to the hospital, due to a sepsis from methicillin-sensitive *Staphylococcus aureus*, an acute kidney injury and worsening of diarrhea (up to 10 bowel movements per day). On admittance, there was no abdominal pain. Weight = 42 Kg, Hb = 9.4 g/dl, CRP = 4.6 mg/dl, CDAI = 183. She was treated with parenteral feeding, antibiotic therapy (rifampicin and levofloxacin) and nabilone (1 mg per day), considering her severe malnutrition. She had watery stool and about 10 bowel movements per day. Concurrent medications included kaolin, loperamide (12 mg, daily) and nutritional supplement. After 3 months, nabilone was discontinued; she had only one bowel movement per day, without blood or mucus in the stools (weight = 45.5 kg). The symptoms did not reappear in the following three months. She had 4 bowel movements per day, with semi-solid stools, no evidence of blood or mucus. Weight = 45 kg. No adverse events were reported during and after nabilone treatment.

### Patient 3

She is a 75-year-old, white female, with a history of melanoma resected from her right leg in 1989 and reactivated to inguinal lymph nodes in 2012. In November 2013, the patient underwent a spleno-pancreatic resection, due to pancreatic cancer; the post-operative course was complexed due to an entero-pancreatic fistula and intestinal obstruction. From March 2014, the patient began to complain of post-prandial diarrhea, not present when the patient was fasting. Colonoscopy did not show mucosal alterations, loperamide (2 mg, as needed) and pancreatic enzymes were not effective. In March 2015, a computerized tomography (CT) scan was performed, with no evidence of abdominal recurrence of melanoma. With the medical prescription of mesalazine and budesonide, in April 2015, the patient had a general improvement, the diarrheal symptoms decreased, and the results of stool cultures were negative. In October 2015, a metastatic pulmonary nodule was removed; the patient began chemotherapy the following month (dabrafenib, 300 mg daily), together with painkillers. Diarrhea reappeared, the patient had poor appetite (weight = 38 kg), therefore she started to take nabilone (1 mg/day) in April 2016. Concurrent medications included pregabalin (150 mg daily), dabrafenib (300 mg daily), trametinib (2 mg daily), tramadol (150 mg daily), budesonide (6 mg daily), rabeprazol (10 mg daily), mesalazine and pancreatic enzymes. Nabilone treatment lasted three months, the patient improved, and the diarrheal symptoms. Weight post-nabilone = 38 kg. No side effects were reported during and after treatment. Three months after the end of the therapy, the benefits remained.

### Patient 4

Patient 4 is a 40-year-old, white male, afflicted by the consequences of an extended mesenteric thrombosis. On admittance, he also complained of pre-hepatic portal hypertension, cavernous transformation of the portal vein and esophageal varices (grade F1). In the history, the patient suffered from chronic hepatitis C virus infection. Diarrhea began in March 2013, the patient was suffering simultaneously from malabsorption (hypoproteinaemia with low albumin levels, hyposideraemia and decreased levels of pseudocholinesterase). Screening for celiac disease was negative, colonoscopy revealed no pathologies in act. There were no intestinal infections and no histologic or endoscopic features of IBD. Despite the introduction of mesalazine and steroids, the patient suffered from 8 bowel movements per day. For severe diarrhea and malabsorption, we started administering nabilone (1 mg/day) in June 2013 (Hb = 9.2 g/dl, platelets = 548,000/mmc, white blood cells or WBC = 4890/mmc, CRP < 0.2 mg/dl). Weight = 46 Kg. Concomitant medications included lansoprazole (60 mg, daily), levosulpiride (50 mg, daily), pregabalin (150 mg, daily), mesalazine (3200 mg daily), low molecular weight heparin (8000 U daily) and oxycodone (5 mg as needed). The therapy was discontinued in July 2013, the patient interrupted autonomously the intake of nabilone because of severe fatigue and mental confusion. At the end of the therapy, the patient reported 2–3 bowel movement per day, without blood or mucus (CRP < 0,2 mg/dl, no leucocytosis or piastrinosis). Weight = 49 kg. Side effects disappeared in the next three months, the patient reported 1 bowel movement per day (weight = 48.5 kg). Concomitant medications were unaltered. A second nabilone administration was denied to the patient due to the risk of adverse events.

### Patient 5

She is a 71-year-old, white female, affected by systemic sclerosis. The diagnosis was done in June 2010, the first symptoms reported were polyarthritis and the Raynaud’s phenomenon (antinuclear antibody and rheumatoid factor tests positive). In that episode, the patient also reported GI bleeding due to a gastric antral vascular ectasia, treated with argon plasma coagulation and other medicines (levonorgestrel/etinilestradiol and octreotide), with a partial response. In August 2012, colonoscopy revealed the presence of inflammation and substenosis in the sigmoid colon. Diarrhea started in January 2015, she complained of 4 liquid feces evacuations per day, without the presence of blood, despite the simultaneous assumption of rifaximin, kaolin and lactic ferments. In August 2015, she was admitted to hospital because of severe diarrhea (10 liquid feces evacuations per day) and malabsorption with hypoalbuminemia, decreased levels of pseudocholinesterase (PCHE = 3071 UI/l), hypokalemia, hypocalcemia (blood calcium = 7.5 mg/dl), leucocytosis and mild anemia (WBC = 14,290/mmc, Hb = 12.1 g/dl). Weight = 45 Kg. Blood magnesium = 1.1 mg/dl, serum iron = 28 mcg/dl, serum ferritin = 24 ng/ml, CRP < 0.2 mg/dl. This episode was treated with rifaximin, mesalazine and budesonide, with a general improvement, but without controlling the diarrheal symptoms. The introduction of nabilone (1 mg daily, for five days, then 1 mg on alternate days) immediately improved the symptoms. Upon discharge the patient reported 3 evacuations per day, with semi-solid stools. Concomitant medications included levotiroxin (200 mcg daily), budesonide (9 mg daily, decreasing gradually over the following three months), rifaximin (800 mg daily for seven days a month), lansoprazole (60 mg daily), furosemide (25 mg daily) and tramadol (15 drops as needed). In September 2015, the patient reported a worsening of diarrhea; the clinical situation improved with increasing the dosage of nabilone (1 mg daily). Weight = 49 Kg. Daily bowel movements = 4. Nabilone therapy finished in November 2015. After three months the patient was stable, she had no abdominal pain, and reported 3 evacuations per day, without blood or mucus (weight = 50 Kg). No side effects were reported.

### Patient 6

She is a 47-year-old, white female, affected by short bowel syndrome and chronic diarrhea, which had occurred after ileal and colonic resection. Diarrhea began in April 2008, after a surgical intervention, due to an intestinal obstruction. Histological investigations identified a tubular adenoma (dysplasia: low grade). In her history, the patient had undergone hysterectomy and bilateral annessiectomy because of uterine cancer, followed by radiotherapy. In 2006, she underwent a left unilateral nephrectomy, due to an adhesiolysis. On admittance, in March 2009, she had 8 bowel movements per day, no sign of inflammation (WBC = 7560/mmc, platelets = 184,000/mmc, CRP = 1.94 mg/dl) and nutritional values unaltered (serum proteins = 7.0 g/dl, serum iron = 48 mcg/dl, serum vitamin B12 = 222 pg/dl, serum folic acid = 11.60 mg/dl, serum pre-albumin = 20.0 mg/dl). Weight = 38 Kg. Having excluded gastro-intestinal infections and celiac disease, a high-calorie diet was started. Colonoscopy revealed no sign of inflammation and regular anastomosis. Initially, diarrhea was treated with loperamide (2 mg, as needed) and kaolin, with no benefit. Later, also octreotide therapy (0.1 mg, subcutaneously) failed to relieve diarrheal symptoms. Nabilone treatment (1 mg daily) lasted three months and proved to have partial control of symptoms (5 bowel movements per day). Concomitant medications included fosinopril (20 mg daily), paroxetine (20 mg daily), alprazolam (1 mg daily), lormetazepam (2 mg daily), alendronate (70 mg weekly), kaolin (two daily doses) and loperamide (8 mg daily). In June 2009, she had no abdominal pain but reported 5 bowel movements per day. Weight = 39 Kg. After three months, the patient continued to report 5 bowel movements/daily, she also reduced the loperamide dosage (2 mg daily). Weight = 37.5 Kg. No side effect and/or signs of addiction were reported during and after nabilone treatment.

## Discussion and conclusions

We report the progression of six cases of chronic diarrhea treated with an oral CB1-receptor agonist, previously managed with etiologic and symptomatic drugs, without benefits. Most of them used antibiotics and steroids without benefit; others did not improve with the nonspecific antidiarrheal therapy (loperamide and kaolin). The sample of patients was heterogeneous, considering the etiology; they were suffering from different GI disorders, chronic diarrhea was their common symptom. All patients complained of a poor quality of life and other symptoms, such as pain, fatigue, appetite loss and dehydration. Accordingly, it was decided to prescribe nabilone for the evidence linking the endocannabinoid system and pain, regulation of appetite and intestinal motility [28], when no other treatment was available. It was administered daily as a compassionate drug, for three months. The main outcome was focused on the safety and efficacy of oral nabilone, the number of bowel movements and the change in body weight were the parameters considered before and after therapy.

After three months, nabilone improved the health of almost every patient (data are reported in Table 2). The daily bowel movements dropped by over 50% and the patients gained weight; the number of antidiarrheal medicines decreased for many of them. Three months after the end of the therapy, the improvements were essentially maintained. In one case, a patient kept the number of daily bowel movements, whereas the body weight was stable, but he reported to feel better. Current results speculate about cannabis and cannabinoids as an attractive approach for chronic GI disorders presenting abdominal pain, inflammation and/or hypermotility, when conventional treatments are not effective. An interesting aspect of this study consisted of the use of a synthetic cannabinoid, a selective agonist for CB1 receptors. The daily administration of nabilone has made possible to highlight only the CB1-mediated effects, emphasizing their importance in inhibiting GI motility and transit [29], and stimulating appetite [30, 31] both short- and long-term. To date, only another study has studied the impact of a CB1 receptor agonist on colonic transit, in irritable bowel syndrome patients [32]. The results were not entirely satisfactory, probably the duration of the treatment was too short.

**Table 2:** Retrospective results of nabilone in the treatment of refractory chronic diarrhea

	Pre-nabilone	Post-nabilone	Follow-up (3 months)
Daily bowel movements (mean ± SD)	7,5 ± 2,66	2,25 ± 1,94	2,5 ± 1,97
Weight (mean ± SD)	43,2 ± 4,75	45,4 ± 5,75	45,2 ± 6,19

*SD* standard deviation

As far as the safety and tolerability are concerned, oral nabilone was well tolerated by almost all patients. In addition, it is important to note that five out of six patients were taking at the same time opiates, anticonvulsants and/or benzodiazepines. A single patient interrupted nabilone after one month, due to severe fatigue and mental confusion; the symptoms disappeared over the next three months. Together, he assumed pregabalin (150 mg/daily) and oxycodone (5 mg as needed). For some years, the patient had previously suffered from a generalized anxiety disorder. In some cases, the use of cannabinoids may unmask psychiatric symptoms, especially in individuals receiving psychoactive drugs, as well as in patients with concomitant manic-depressive illnesses, schizophrenia, anxiety and depression. Accordingly, the benefit/risk ratio of nabilone use should be carefully evaluated case by case. In this patient, the adverse event was transient, and a second administration was denied.

To the best of our knowledge, this is the first study that provide preliminary evidence that a CB1-receptor agonist may be safe and play a beneficial role when the outcomes are focused on chronic GI disorders, especially on the diarrheal symptom. Moreover, even though the duration of the therapy did not exceed three months, improvements were maintained over time. Despite all, we are still a long way from the answers about the role of cannabis in GI disorders, because of the limited generalizability of this study and the mixed retrospective results in the medical literature, often due to the wide variety of doses and formulations of cannabis used, as well as the lack of randomized clinical trials [33–35].

Our approach benefits from evaluating CB1-receptor mediated outcomes in clinical practice, in a small population with multifaceted problems, thereby reflecting real adherence to treatment/intervention. However, it also has some limitations; only six patients were evaluated, a contemporaneous placebo treatment group was missing, and the duration of treatment was only three months. These outcomes encourage the study of cannabinoids acting on CB1 receptors in chronic GI disorder, offering a chance for a significant improvement in the quality of life of selected patients when traditional approaches are not beneficial.

## Data Availability

The datasets used and analysed during the current study are available from the corresponding author on reasonable request.

## Abbreviations

CB1: G protein-coupled cannabinoid receptors 1
CB2: G protein-coupled cannabinoid receptors 2
CDAI: Crohn’s Disease Activity Index
CRP: C-reactive protein
CT: Computerized tomography
ECS: Endocannabinoid system
ENS: Enteric nervous system
ESR: Erythrocyte Sedimentation Rate
GI: Gastrointestinal
Hb: Haemoglobin
Ht: Haematocrit
IBD: Inflammatory bowel disease
NSAIDs: Non-steroidal anti-inflammatory drugs
PCHE: Pseudocholinesterase
PPARs: Peroxisome proliferator-activated receptors
TRPV: Transient receptor potential cation channels
WBC: White blood cells
Δ^9^-THC: Δ^9^-tetrahydrocannabinol
